# Effects of Metabolic Syndrome and its components on the postoperative recurrence in Chronic Rhinosinusitis with Nasal Polyps’ patients

**DOI:** 10.1016/j.bjorl.2023.101371

**Published:** 2023-12-05

**Authors:** Yu Chen, Tiansheng Wang, Ru Gao, Fengjun Wang

**Affiliations:** a Central South University, The Third Xiangya Hospital, Department of Otolaryngology Head and Neck Surgery, Changsha, China; b Xiangya Hospital of Central South University, Department of Otolaryngology Head and Neck Surgery, Changsha, China; c Xiangya Hospital of Central South University, Hunan Province Key Laboratory of Otolaryngology Critical Diseases, Changsha, China; d Xiangya Hospital of Central South University, National Clinical Research Center for Geriatric Disorders, Changsha, China

**Keywords:** Chronic Rhinosinusitis with Nasal Polyps, Metabolic Syndrome, Functional endoscopic sinus surgery, Prognosis, Recurrence

## Abstract

**Objectives:**

Metabolic Syndrome (MetS) has been established as a significant factor in the pathogenesis of numerous chronic inflammatory conditions. However, its role in Chronic Rhinosinusitis with Nasal Polyps (CRSwNP) is unknown. This study aims to investigate the association between MetS, its components, and the risk of postoperative recurrence in Chinese patients with CRSwNP.

**Methods:**

A retrospective cohort study was conducted on CRSwNP patients who underwent endoscopic sinus surgery in our hospital. Patients were divided into MetS and non-MetS groups, and the clinical characteristics and recurrence rates were compared. All CRSwNP patients were followed up for more than 2-years and further categorized into non-recurrent and recurrent groups. Binary logistic regression analyses were performed to examine the effects of MetS and its components on the risk of postoperative recurrence.

**Results:**

A total of 555 CRSwNP patients were enrolled in the present study, 157 patients were included in the MetS group and 398 patients were categorized into the non-MetS group. The recurrence rate in the MetS group was significantly higher compared to the non-MetS group (*p* < 0.05). The rate of MetS, overweight or obesity, hyperglycemia and dyslipidemia were higher in the recurrent group in comparison with the non-recurrent group (*p* < 0.05). Multivariate logistic regression analysis suggested that MetS, overweight or obesity, hyperglycemia, dyslipidemia, and accompanying allergic rhinitis were associated with the risk of postoperative recurrence of CRSwNP (*p* < 0.05). Moreover, adjusted and unadjusted regression models showed that MetS was an independent risk factor for postoperative recurrence of CRSwNP, and the risk increased with more components of MetS included (*p* < 0.05).

**Conclusion:**

Our findings revealed that MetS independently increased the risk of postoperative recurrence in patients with CRSwNP, with the risk escalating as the number of MetS components increased. Moreover, accompanying allergic rhinitis was also demonstrated to be a potential risk factor for CRSwNP recurrence.

**Level of evidence:**

Level 4.

## Highlights

• MetS increased the risk of postoperative recurrence in CRSwNP.
• Recurrence risk increases with an increasing number of components.
• Allergic rhinitis as a potential risk factor for CRSwNP recurrence.

## Introduction

Chronic Rhinosinusitis (CRS) is a chronic inflammatory disease that occurs in the mucous of the nasal cavity and sinuses.^1^ The representative symptoms are nasal congestion, mucopurulent nasal discharge, headache, and loss of smell.^2^ CRS can be clinically classified into two phenotypes: Chronic Rhinosinusitis without Nasal Polyps (CRSsNP) and Chronic Rhinosinusitis with Nasal Polyps (CRSwNP).^3^ These two phenotypes exhibit different clinical features and intrinsic pathophysiological mechanisms, and CRSwNP is regarded to present more complex etiologies and poorer prognosis.^2^

In recent years, Endoscopic Sinus Surgery (ESS) has become the primary treatment modality for patients with CRSwNP who show inadequate responses to medical therapy, and its effectiveness and safety have garnered widespread acceptance.^4^ The primary objective of ESS is to eliminate permanent nasal and sinus lesions, restore nasal and sinus ventilation and drainage, reduce mucosal inflammation, and facilitate the recovery of mucosal glandular secretion and ciliary clearance function.^5^ Although ESS can effectively relieve symptoms and enhance patients’ quality of life, real-world studies have reported an increasing number of CRSwNP patients experiencing postoperative inflammatory relapses and recurrences, with recurrence rates ranging from approximately 10%–79%.^5^, ^6^ Hence, investigating the risk factors for postoperative recurrence in CRSwNP and devising personalized and precise treatment strategies represent pressing challenges.

Metabolic Syndrome (MetS) is characterized by metabolic abnormalities, which include central obesity, elevated blood pressure, glucose intolerance, and dyslipidemia, with insulin resistance as a central underlying factor.^7^ The precise mechanisms responsible for MetS remain incompletely elucidated. Nevertheless, central obesity is acknowledged as a key component and an initiating factor for MetS. Central obesity can trigger insulin resistance, which, in turn, disrupts glucose and lipid metabolism, and leads to elevated blood pressure.^8^, ^9^ Recently, the rising intake of surplus calories, decreased physical activity, and the adoption of unhealthy lifestyles have collectively led to an increased prevalence of MetS. Consequently, MetS has emerged as a growing global public health issue.^10^, ^11^ According to estimates provided by the International Diabetes Federation, approximately 25% of the world’s population suffers from MetS, and the prevalence in China has reached 20%–35% and continues to increase.^11^ Previous studies found that MetS significantly increased the incidence of various diseases including asthma,^12^ psoriases,^13^ atopic dermatitis,^14^ and systemic lupus erythematosus,^15^ and was significantly associated with poor disease prognosis. However, our understanding of the role of MetS and its individual components in the pathophysiology of CRSwNP is currently lacking. The influence of MetS and its constituent factors on the risk of postoperative recurrence in CRSwNP patients remains uncertain. Further investigations are required to unravel potential connections and assess the impact of MetS on CRSwNP recurrence.

The primary objectives of this study were to examine the influence of MetS and its components on the risk of postoperative recurrence in CRSwNP patients and to explore other potential risk factors associated with postoperative recurrence.

## Methods

### Study design, setting, and participants

This study retrospectively analyzed the clinical data of patients with CRSwNP who treated with ESS in our institution between January 2017 and March 2022. All patients underwent detailed medical history inquiry and recording, nasal endoscopy examination, laboratory examination, and sinus Computed Tomography (CT). The inclusion criteria were as follows: 1) >18 years old, 2) complete clinical data; 3) no previous history of ESS; 4) conform to the guidelines for the diagnosis and treatment of CRSwNP.^16^ The exclusion criteria were as follows: 1) combined tumor or other serious disease; 2) psychiatric abnormality; 3) drop-out during following-up; 4) fungal sinusitis, antrochoanal polyp, and cystic fibrosis.

After ESS, CRSwNP patients were advised to follow a treatment regimen comprising daily nasal saline irrigation, antibiotics, topical corticosteroids, and periodic endoscopic debridement. All participants routinely used mometasone furoate aqueous nasal spray after surgery, applying approximately 100 μg per nostril once daily. They were scheduled for regular follow-up appointments where their progress was monitored through endoscopic examinations. Recurrence was defined by reappeared clinical symptoms and endoscopic and CT evidence for at least 2 months despite the rescue regimen of antibiotics and oral steroids as previously described.^17^ After a minimum follow-up period of 2 years, the patients were divided into two groups based on their postoperative outcomes: the recurrent group and the non-recurrent group. This retrospective study was approved by the Human Ethical Committee of Xiangya Hospital of Central South University (202204199). Since the study did not involve any private patient information or commercial interests, it was determined to be exempt from the requirement of informed consent by the Ethics Committee.

### Data collection

Data were extracted from patient’s medical files and collected in a secure health database. Baseline data include age, gender, smoking, alcohol consumption, duration of disease, allergic rhinitis, asthma, Body Mass Index (BMI), Fasting Blood Glucose (FBG), Systolic Blood Pressure (SBP), Diastolic Blood Pressure (DBP), Total Cholesterol (TC), Low-Density Lipoprotein-Cholesterol (LDL-C), Triglyceride (TG), High-Density Lipoprotein-Cholesterol (HDL-C). Biochemical data including FBG, TG, and HDL-C are routinely measured on the plasma of fasted individuals. Medical history data including diabetes mellitus, hypertension, and dyslipidemia were self-reported and confirmed by physicians.

### Definition of MetS

MetS was diagnosed according to the criteria of the Chinese Diabetes Society.^18^ The diagnosis of MetS was made when a patient met three or more of the following conditions or components: 1) overweight or obesity: BMI ≥ 25.0 kg/m^2^; 2) hyperglycemia: fasting blood glucose ≥6.1 mmoL/L and/or 2-h postprandial blood glucose ≥7.8 mmoL/L or drug treatment for any type of diabetes mellitus; 3) hypertension: blood pressure ≥40/90 mmHg, or (and) hypertension were confirmed and being treated; 4) dyslipidemia: fasting triglycerides ≥1.70 mmoL/L and/or HDL-C < 0.9 mmoL/L for males and <1.0 mmoL/L for females.

### Statistical analysis

Categorical variables are expressed as numbers and percentages and compared utilizing the Chi-Squared test. Quantitative variables with normal distribution are shown as mean ± standard deviation and compared with the Student’s *t*-test between the two groups. Those data without normal distribution are presented as median and Interquartile Ranges (IQRs), and the Mann–Whitney *U* test is utilized in the comparison between the two groups. Patients are categorized into non-recurrent CRSwNP and recurrent CRSwNP groups. Univariate and multivariate regression analyses are performed to explore the associations between MetS and its components and the risk of CRSwNP recurrence in different adjusted models. Statistical significance is regarded as a two-tailed *p* < 0.05.

## Results

### Characteristics and baseline data of the study subjects

After 2 years follow-up, a total of 555 patients with CRSwNP, including 365 males and 190 females, were included in this study. Among them, 323 patients were divided into the non-recurrent group and 232 patients were included in the recurrent group. The mean age of the patients is 43.7 years, and the mean duration of the disease is 65.7 months. Among them, 157 patients are confirmed to have MetS. The clinical data of the study subjects are shown in Table 1 in detail.

**Table 1:** Clinical characteristics of all included CRSwNP patients.

**Variables**	
Number of enrolled patients	555
Age, years	43.7 (31.0–55.0)
Male, n (%)	365 (65.8)
Smoking, n (%)	90 (16.2)
Alcohol consumption, n (%)	45 (8.1)
Duration of disease, months	65.7 (12.0–96.0)
Allergic rhinitis, n (%)	149 (26.9)
Asthma, n (%)	40 (7.2)
BMI, kg/m^2^	24.0 (21.4–26.4)
FBG, mmoL/L	5.2 (4.6–5.4)
TC, mmoL/L	4.7 (4.2–5.5)
LDL-C, mmoL/L	31 (2.6–3.5)
SBP, mmHg	125.9 (114.0–134.0)
DBP, mmHg	79.5 (72.0–85.0)
TG, mmoL/L	1.6 (1.0–1.9)
HDL-C, mmoL/L	1.2 (0.9–1.3)
MetS, n (%)	157 (28.3)
**MetS component**	
Overweight or obesity, n (%)	150 (27.0)
Hypertension, n (%),	156 (28.1)
Hyperglycemia, n (%)	158 (28.5)
Dyslipidemia, n (%)	190 (34.2)

CRSwNP, Chronic Rhinosinusitis with Nasal Polyps; BMI, Body Mass Index; FBG, Fasting Blood Glucose; SBP, Systolic Blood Pressure; DBP, Diastolic Blood Pressure; TC, Total Cholesterol; TG, Triglyceride; HDL-C, High Density Lipoprotein-Cholesterol; LDL-C, Low Density Lipoprotein-Cholesterol; MetS, Metabolic Syndrome.

### Clinical characteristics of CRSwNP patients with and without MetS

As displayed in Table 2, 157 cases were divided into the MetS group, and 398 cases were categorized into the non-MetS group. The occurrence of recurrent CRSwNP was significantly higher in the MetS group compared to the non-MetS group (*p* < 0.05). Moreover, in comparison with the non-MetS group, the MetS group exhibited higher levels of various parameters including age, smoking, alcohol consumption, BMI, FBG, SBP, DBP, LDL-C, TG, and rates of overweight or obesity, hypertension, hyperglycemia, and dyslipidemia (*p* < 0.05).

**Table 2:** Baseline clinical characteristics of CRSwNP patients among two groups.

Variables	Non-MetS (n = 398)	MetS (n = 157)	*p*
Age, years	42.7 (30.0–54.0)	52.1 (46.0–63.0)	<0.001
Male, n (%)	265 (66.5)	100 (63.7)	0.552
Smoking, n (%)	51 (12.9)	39 (24.6)	0.001
Alcohol consumption, n (%)	23 (5.8)	22 (14.0)	0.003
Duration of disease, months	63.1 (12.0–84.0)	88.0 (7.0–120.0)	0.417
Allergic rhinitis, n (%)	104 (26.1)	45 (28.7)	0.595
Asthma, n (%)	29 (7.3)	11 (7.0)	1.000
BMI, kg/m^2^	23.6 (21.2–25.5)	27.5 (25.5–28.7)	<0.001
FBG, mmoL/L	5.0 (4.6–5.3)	6.4 (4.9–7.4)	<0.001
SBP, mmHg	123.8 (113.0–132.0)	144.1 (130.0–159.0)	<0.001
DBP, mmHg	78.3 (72.0–84.0)	89.8 (79.5–100.0)	<0.001
TC, mmoL/L	4.8 (4.2–5.4)	4.8 (4.1–5.5)	0.858
LDL-C, mmoL/L	2.9 (2.5–3.3)	3.2 (2.7–3.7)	0.002
TG, mmoL/L	1.5 (1.0–1.7)	2.7 (1.8–3.1)	<0.001
HDL-C, mmoL/L	1.2 (1.0–1.3)	1.0 (0.8–1.1)	<0.001
MetS component			
Overweight or obesity, n (%)	57 (14.3)	93 (59.2)	<0.001
Hypertension, n (%)	57 (14.3)	99 (63.1)	<0.001
Hyperglycemia, n (%)	42 (10.6)	116 (73.9)	<0.001
Dyslipidemia, n (%)	76 (19.1)	114 (72.6)	<0.001
Recurrent CRSwNP, n (%)			<0.001
Yes	146 (36.7)	86 (54.8)	
No	252 (63.3)	71 (45.2)	

CRSwNP, Chronic Rhinosinusitis With Nasal Polyps; MetS, Metabolic Syndrome; BMI, Body Mass Index; FBG, Fasting Blood Glucose; SBP, Systolic Blood Pressure; DBP, Diastolic Blood Pressure; TG, Triglyceride; HDL-C, High Density Lipoprotein-Cholesterol.

### Univariate analysis of risk factors for recurrence in CRSwNP patients

The participants were further divided into the non-recurrent CRSwNP group and the recurrent CRSwNP group. The demographic and clinical data of these two groups are compared in Table 3. Age, duration of disease, BMI, FBG, TC, TG, HDL-C, and allergic rhinitis concomitant rates were increased in the recurrent CRSwNP group compared to the non-recurrent CRSwNP group (*p* < 0.05). Interestingly, upon comparing the prevalence of MetS and its individual components between the recurrent CRSwNP and non-recurrent CRSwNP groups, we found that the rates of overweight or obesity, hyperglycemia, and dyslipidemia were significantly higher in the recurrent CRSwNP group (*p* < 0.05). However, there was no significant difference in the occurrence of elevated blood pressure between the two groups (*p* > 0.05).

**Table 3:** Comparison of clinical data between non-recurrent and recurrent groups.

Variables	Primary CRSwNP (n = 323)	Recurrent CRSwNP (n = 232)	*p*
Age, years	42.5 (30.0–54.0)	45.5 (33.0–56.0)	0.014
Male, n (%)	203 (62.9)	162 (69.8)	0.103
Smoking, n (%)	51 (15.8)	39 (16.8)	0.816
Alcohol consumption, n (%)	20 (6.1)	25 (10.8)	0.059
Duration of disease, months	50.8 (10.5–72.1)	89.9 (12.0–120.0)	<0.001
Allergic rhinitis, n (%)	69 (21.4)	80 (34.6)	0.001
Asthma, n (%)	18 (5.5)	22 (9.5)	0.096
BMI, kg/m^2^	23.2 (20.7–25.3)	25.3 (23.0–27.5)	<0.001
FBG, mmoL/L	5.0 (4.5–5.3)	5.4 (4.6–5.5)	0.005
SBP, mmHg	125.3 (115.0–134.0)	126.2 (114.0–135.0)	0.708
DBP, mmHg	78.9 (73.0–83.0)	79.8 (72.0–87.0)	0.391
TC, mmoL/L	4.7 (4.1–5.3)	5.0 (4.3–5.6)	0.001
LDL-C, mmoL/L	2.9 (2.5–3.3)	3.0 (2.5–3.6)	0.131
TG, mmoL/L	1.5 (0.9–1.9)	1.7 (1.1–2.1)	<0.001
HDL-C, mmoL/L	1.2 (1.0–1.3)	1.1 (0.9–1.2)	<0.001
MetS, n (%)	71 (22.0)	86 (37.1)	
MetS components			
Overweight or obesity, n (%)	53 (16.4)	97 (41.8)	<0.001
Hypertension, n (%)	89 (27.6)	67 (28.8)	0.774
Hyperglycemia, n (%)	63 (19.5)	95 (40.9)	<0.001
Dyslipidemia	84 (26.0)	106 (45.7)	<0.001

CRSwNP, Chronic Rhinosinusitis with Nasal Polyps; BMI, Body Mass Index; FBG, Fasting Blood Glucose; SBP, Systolic Blood Pressure; DBP, Diastolic Blood Pressure; TC, Total Cholesterol; LDL-C, Low Density Lipoprotein-Cholesterol; TG, Triglyceride; HDL-C, High density Lipoprotein-Cholesterol; MetS, Metabolic Syndrome.

### Binary logistic regression analysis of potential factors for postoperative recurrence

Variables with significant differences in Table 3 were further included in the multivariate logistic regression analysis. The results in Table 4 demonstrated that MetS was significantly associated with the risk of postoperative recurrence of CRSwNP (OR = 2.370, *p* < 0.001). Additionally, allergic rhinitis (OR = 2.630, *p* < 0.001), overweight or obesity (OR = 1.218, *p* < 0.001), hyperglycemia (OR = 1.931, *p* < 0.001), and dyslipidemia (OR = 1.795, *p* < 0.001) were potential risk factors for postoperative recurrence of CRSwNP.

**Table 4:** Multivariate binary logistic regression analysis for CRSwNP recurrence.

Variables	Multivariate		
	OR	95% CI	*p*
Age, years	1.014	0.989‒1.028	0.095
Duration of disease, months	1.005	0.997‒1.006	0.103
Allergic rhinitis			
Yes	2.630	1.636‒4.228	<0.001
No (ref.)			
Overweight or obesity			
Yes	1.218	1.147‒1.293	<0.001
No (ref.)			
Hyperglycemia			
Yes	1.931	1.111‒3.355	<0.001
No (ref.)			
Dyslipidemia			
Yes	1.795	1.283‒3.143	<0.001
No (ref.)			
MetS			
Yes	2.370	1.298‒4.328	<0.001
No (ref.)			

CRSwNP, Chronic Rhinosinusitis with Nasal Polyps; OR, Odds Rate; CI, Confidence Interval; MetS, Metabolic Syndrome.

To investigate the impact of MetS and its components on the risk of CRSwNP recurrence, we performed both unadjusted and adjusted logistic regression analyses. These analyses included adjustments for several potential confounders such as age, gender, smoking, duration of disease, alcohol consumption, allergic rhinitis, and asthma. The results from various adjustment models presented in Table 5 indicated that the presence of MetS substantially elevated the risk of postoperative recurrence in CRSwNP. Notably, this association remained statistically significant even after adjusting for potential confounding variables (*p* < 0.05). Furthermore, it was observed that the risk of postoperative recurrence in CRSwNP increased as more components of MetS were included (*p* < 0.05).

**Table 5:** Risk of CRSwNP recurrence based on different MetS component.

	Non-MetS (component < 3)	OR (95% CI) (component = 3)	*p*	OR (95% CI) (component = 4)	*p*
**Unadjusted**	Ref.	2.027 (1.163–3.535)	<0.001	2.645 (1.124–6.224)	<0.001
**Model 1**	Ref.	2.244 (1.273–3.953)	0.005	2.805 (1.185–6.639)	0.019
**Model 2**	Ref.	2.235 (1.237–4.038)	0.008	3.237 (1.330–7.878)	0.010
**Model 3**	Ref.	2.331 (1.269–4.284)	0.006	3.012 (1.210–7.496)	0.018

Model 1: adjusted for age and gender.

Model 2: adjusted for age and gender, smoking, and alcohol consumption.

Model 3: adjusted for age and gender, smoking, alcohol consumption, duration of disease, allergic rhinitis, and asthma.

CRSwNP, Chronic Rhinosinusitis with Nasal Polyps; MetS, Metabolic Syndrome; OR, Odds Rate; CI, Confidence Interval.

## Discussion

Although ESS is widely acknowledged for its effectiveness and safety in managing CRSwNP, a considerable proportion of patients continue to grapple with recurrence. This challenge can be attributed to the intricate and not fully elucidated etiology of CRSwNP, compounded by the high degree of variability within the disease.^19^, ^20^ Therefore, it is essential to conduct a comprehensive assessment of the clinical characteristics of CRSwNP within distinct patient populations and delve into the multiple factors that impact prognosis and the likelihood of postoperative recurrence. This approach facilitates the development of customized interventions and individualized treatment strategies. In this study, we aimed to examine the relationship between MetS and its individual components and the likelihood of postoperative recurrence in individuals with CRSwNP. Our results indicated a substantial link between MetS and an elevated risk of postoperative recurrence in CRSwNP patients, with the risk rising in tandem with an increasing number of MetS components. Furthermore, we identified coexisting allergic rhinitis as a potential risk factor for recurrence.

MetS has become a significant global health concern, affecting approximately 20%–30% of the population. Its impact on inflammatory diseases, cardiovascular diseases, and tumors is gaining considerable attention in terms of both pathogenesis and prognosis.^21^, ^22^, ^23^, ^24^ Despite the increasing recognition of the impact of MetS on various diseases, there is a scarcity of research investigating its role in CRSwNP. Prior research has predominantly concentrated on investigating the relationship between specific components of MetS and the development of CRSwNP. For instance, Steele et al.^25^ established an elevated risk of CRS in overweight and obese individuals, while another study emphasized a significant association between diabetes mellitus and the incidence of CRSwNP. In this context, hyperglycemia was found to exacerbate olfactory dysfunction in CRS patients.^26^ Although these studies have shed light on the link between individual MetS components and CRS, the effects of MetS on the prognosis and postoperative recurrence of CRSwNP remain largely unexplored.

In our present study, we aim to underscore the previously underemphasized heightened risk of postoperative recurrence in CRSwNP patients with MetS. Furthermore, we investigate the correlation between the number of MetS components and the risk of recurrence. Our findings shed light on MetS and specific MetS components, such as overweight or obesity, hyperglycemia, and dyslipidemia, as factors linked to an increased risk of postoperative recurrence in CRSwNP. It is noteworthy that accumulating evidence indicates that recurrent CRSwNP is characterized by elevated infiltration of inflammatory cells, heightened levels of oxidative stress, and aggravated tissue remodeling.^27^ MetS comprises a constellation of metabolic abnormalities, including central obesity, hyperglycemia, dyslipidemia, and hypertension. These components collectively contribute to more complex pathogenic and prognostic effects.^8^, ^10^ In our hypothesis, we suggest that MetS triggers multiple interlinked pathophysiological mechanisms that intensify both local and systemic inflammation and oxidative stress in CRSwNP. These processes subsequently facilitate the recurrence of CRSwNP following surgery. It is well-established that adipose tissue serves as a key endocrine organ, secreting various adipokines, growth factors, cytokines, and chemokines.^28^ Overweight and obesity have been shown to disrupt adipose tissue function, leading to the production of abnormal immune factors. This includes the activation of macrophages and dysregulation of their polarization, resulting in increased inflammation and significant tissue fibrosis.^29^ Research suggests that high lipid levels can activate the complement system and disrupt the innate immune response, leading to sustained chronic inflammation and structural remodeling of nasal mucosal tissue through epithelial-mesenchymal transformation.^30^, ^31^, ^32^ Dyslipidemia has also been significantly associated with inflammatory response status, oxidative stress levels, and pro-fibrotic processes, which play a role in the underlying mechanisms of recurrent CRSwNP.^33^ Moreover, chronic hyperglycemia can alter cellular energy metabolism and cause oxidative stress damage, leading to mucosal epithelial barrier dysfunction.^34^ Impaired barrier function allows pathogenic microorganisms to stimulate nasal mucosal epithelial cells to produce inflammatory mediators, activating and recruiting various immune and inflammatory cells, including eosinophils, thus triggering an inflammatory cascade, and exacerbating histopathological changes in CRSwNP.^35^, ^36^ The expressions of systemic inflammation biomarkers, such as CRP, fibrinogen, IL-6, and TNF-α, were found to be enhanced in the serum of MetS patients.^37^ Among them, increased levels of TNF-α can induce the production of inflammatory cytokines, driving inflammation progression and exacerbating tissue remodeling in nasal mucosal tissue.^38^ Therefore, it is reasonable to MetS may play a role in the advancement and outlook of CRSwNP by means of multiple interconnected components. The cumulative impact of incorporating more components could potentially worsen the condition. Nonetheless, the precise mechanisms underpinning the connection between MetS and the postoperative recurrence of CRSwNP necessitate additional investigation and validation.

Furthermore, this study identified a significant association between the prevalence of allergic rhinitis and the recurrence of CRSwNP after surgery. Allergic rhinitis is frequently observed as a coexisting condition in CRSwNP and contributes to the persistent hyper-reactivity of the nasal cavity and sinus mucosa. This hyper-reactive state is characterized by ongoing swelling, heightened exudation, and reduced sinus openings, resulting in a hypoxic environment that promotes the colonization of viruses and bacteria.^39^, ^40^, ^41^ Eosinophils play a critical role in the development of allergic rhinitis and CRSwNP, and tissue eosinophilia is a characteristic feature of both conditions. Eosinophils can contribute to the aggravation of mucosal damage in the respiratory tract, triggering a robust inflammatory response.^40^, ^42^ Moreover, tissue eosinophilia has been linked to more extensive disease in CRSwNP.^43^ The occurrence of an allergic response can stimulate the release of type 2 inflammatory mediators, including IL-4, IL-5, and IL-13, which promote the recruitment and activation of eosinophils, mast cells, and basophils, thereby inducing nasal eosinophilia.^44^ Furthermore, cells in the nasal and paranasal mucosa express surface molecules that attract inflammatory mediators and secrete inflammatory cytokines, creating a feedback loop.^35^ In CRSwNP patients, these adhesion molecules and chemotactic factors are abundantly expressed, potentially aggravate the pathogenesis of CRSwNP, and were believed to underlie the mechanism of allergic rhinitis-mediated CRSwNP recurrence in the context of allergic rhinitis.^45^

This study acknowledges several limitations. Firstly, the recruitment of patients solely from a single medical center in China introduces the possibility of selection bias, which limits the generalizability of the findings to a broader population. Secondly, due to the retrospective nature of the study, it is unable to establish a direct causal relationship between MetS and its components and the recurrence of CRSwNP. Lastly, there could be other unidentified or unmeasured factors that contribute to the risk of postoperative recurrence, such as tissue eosinophilia, which might lead to bias in this study.

## Conclusion

This study represented the first, larger-scale investigation of the clinical features of CRSwNP patients with concurrent MetS. Our findings highlighted a possible connection between MetS and the risk of postoperative recurrence in CRSwNP, with the risk escalating as more MetS components were present. Moreover, we identified allergic rhinitis as a potential risk factor for CRSwNP recurrence. The incorporation of MetS as a novel indicator for postoperative prognostic assessment and risk stratification in CRSwNP patients appears to be clinically meaningful and promising. Nevertheless, to validate and strengthen our conclusions, further high-quality studies are needed.

## Authors’ contributions

Yu Chen contributed to the conception and design of the study. Tiansheng Wang and Yu Gao collected the clinical data and analyzed the data. Fengjun Wang analyzed the results and drafted and corrected the manuscript. All authors contributed to the article and approved the submitted version.

## Funding

No funding.

## Conflicts of interest

The authors declare no conflicts of interest.
